# Alpha-Synuclein Targeting Therapeutics for Parkinson's Disease and Related Synucleinopathies

**DOI:** 10.3389/fneur.2022.852003

**Published:** 2022-05-09

**Authors:** Sindhu Menon, Sabrina Armstrong, Amir Hamzeh, Naomi P. Visanji, Sergio Pablo Sardi, Anurag Tandon

**Affiliations:** ^1^Tanz Centre for Research in Neurodegenerative Diseases, Toronto, ON, Canada; ^2^Department of Laboratory Medicine and Pathobiology, University of Toronto, Toronto, ON, Canada; ^3^Krembil Research Institute, Toronto, ON, Canada; ^4^Sanofi, Framingham, MA, United States; ^5^Department of Medicine, University of Toronto, Toronto, ON, Canada

**Keywords:** Parkinson's disease, prion, gene therapy, anti-aggregation, brain delivery of drugs, immunization

## Abstract

α-Synuclein (asyn) is a key pathogenetic factor in a group of neurodegenerative diseases generically known as synucleinopathies, including Parkinson's disease (PD), dementia with Lewy bodies (DLB) and multiple system atrophy (MSA). Although the initial triggers of pathology and progression are unclear, multiple lines of evidence support therapeutic targeting of asyn in order to limit its prion-like misfolding. Here, we review recent pre-clinical and clinical work that offers promising treatment strategies to sequester, degrade, or silence asyn expression as a means to reduce the levels of seed or substrate. These diverse approaches include removal of aggregated asyn with passive or active immunization or by expression of vectorized antibodies, modulating kinetics of misfolding with small molecule anti-aggregants, lowering asyn gene expression by antisense oligonucleotides or inhibitory RNA, and pharmacological activation of asyn degradation pathways. We also discuss recent technological advances in combining low intensity focused ultrasound with intravenous microbubbles to transiently increase blood-brain barrier permeability for improved brain delivery and target engagement of these large molecule anti-asyn biologics.

## Rationale for Targeting Asyn

Pathological accumulation of alpha-synuclein (asyn) is the common distinguishing trait amongst the group of brain disorders known as synucleinopathies, which include Parkinson's disease (PD), Dementia with Lewy bodies (DLB), and Multiple System Atrophy (MSA). These disorders progressively develop neuronal and glial inclusions enriched in misfolded, phosphorylated, and detergent-insoluble asyn (1, 2). Asyn is a 140 amino acid protein predominantly localized to nerve terminals. Although it lacks a transmembrane domain, its amino-terminal domain can reversibly adopt an amphipathic alpha-helical structure that enables it to partially embed into synaptic vesicle membranes (3). Its localization and behavior during neuronal activity is highly suggestive of a role in vesicle trafficking, though its precise function remains incompletely understood (4). While the unbound, freely-diffusible pool of asyn in cytoplasm is generally thought to be intrinsically disordered, poorly characterized pathological triggers induce rigid beta sheet-enriched conformations that can polymerize into oligomers and fibrils (5). These asyn multimers accumulate for a variety of reasons including superior conformational stability, inefficient removal by protein degradation machinery in neurons, and ongoing self-expansion by interaction with native asyn which then acquires the seed conformation (6).

This process of recruitment and misfolding of native asyn, called permissive templating, is not dependent on cofactors and is readily observed in cell-free conditions with purified recombinant protein. However, the kinetics and range of conformations are constrained by mutations, binding partners, and by oxidative or post-translational modifications. The first evidence of this process in human brain was the recognition that host-to-graft transmission of Lewy bodies (LB) may have occurred in PD patients who had received fetal neuron grafts 10–15 years earlier (7, 8). This seminal observation prompted a paradigm shift in our understanding of the underlying pathophysiological mechanisms and their role in the stereotypical patterns of disease progression previously identified by Braak et al. (1, 9, 10). Subsequent characterizations of asyn internalization, mixing with cellular asyn, and export of misfolded asyn have been replicated in multiple cell and animal models (11). For example, the host-to-graft transmission of cerebral asyn pathology was observed in transgenic asyn mice implanted with neuronal grafts, as well as simpler experimental systems such as co-cultured neurons (12, 13). Furthermore, extracellular seeding with recombinant pre-formed fibrils or synucleinopathy-derived asyn aggregates readily triggers intracellular neuronal inclusions (14–18) and corresponding intracerebral inoculations into transgenic mice expressing human asyn replicate pathological and clinical features of spreading synucleinopathies (19–24).

There is also an additional layer of complexity evident in the differential spatio-temporal patterns of the pathological asyn spread between PD, MSA, and DLB, which imply that each disease develops a unique pattern of neuronal vulnerability. Imaging and post-mortem analyses suggest neuronal connectivity between brain regions is an essential but insufficient predictor of the pathological asyn spread (25). Other local factors such as asyn expression, origin of pathology, and susceptibility to asyn conformational strains play an important role (26–29). Structurally distinct asyn isoforms generated from recombinant proteins have been serially propagated in cells and animals (28, 30, 31), providing unequivocal evidence for prion-like intercellular spread of conformational strains (32).

The multitude of cell and animal models demonstrating intercellular exchange of asyn aggregates have provided a wealth of mechanistic information on asyn internalization and trafficking, and prompted the development and testing of new therapeutic approaches (33, 34). These studies support the idea that overlapping mechanisms exist between the synucleinopathies that initiate and sustain the pathological spread, regardless of the brain regions affected by early pathology or the underlying trigger. Those commonalities offer useful starting points for the development of disease-modifying treatments.

## Biological Pathways That Enable Asyn Pathology Spread

### Mechanisms of Intercellular Asyn Transfer and Consequent Impact on Design of Therapeutics

The possibility that misfolded asyn could be transferred between adjacent neurons and contribute to pathological spread provided the impetus to establish the mechanisms of asyn transfer (35). Extracellular asyn is detectable in saliva, plasma and cerebrospinal fluid suggesting physiological mechanisms to enable its secretion. Intercellular exchange of asyn is complicated by the fact that it is a cytosolic protein, so secretion of asyn or its multimers necessitates passage through one or more lipid bilayers. Asyn has been detected within large intracellular vesicles and a non-traditional calcium-dependent exocytic process has been proposed (36–40). Some asyn may also escape due to neuronal injury or death, though the contribution of bolus asyn release is likely minor with predominantly local effects (12). For example, neuronal injury and degeneration in rats injected with AAV containing human asyn was accompanied by a substantial reduction of caudo-rostral asyn propagation suggesting that transmission is preferentially mediated by an active mechanism (41). Anatomical evaluation of spreading pathology following seeding with asyn fibrils also implicate neuronal connectivity as a key determinant of its spread across distal brain regions (29).

**Figure 1:**
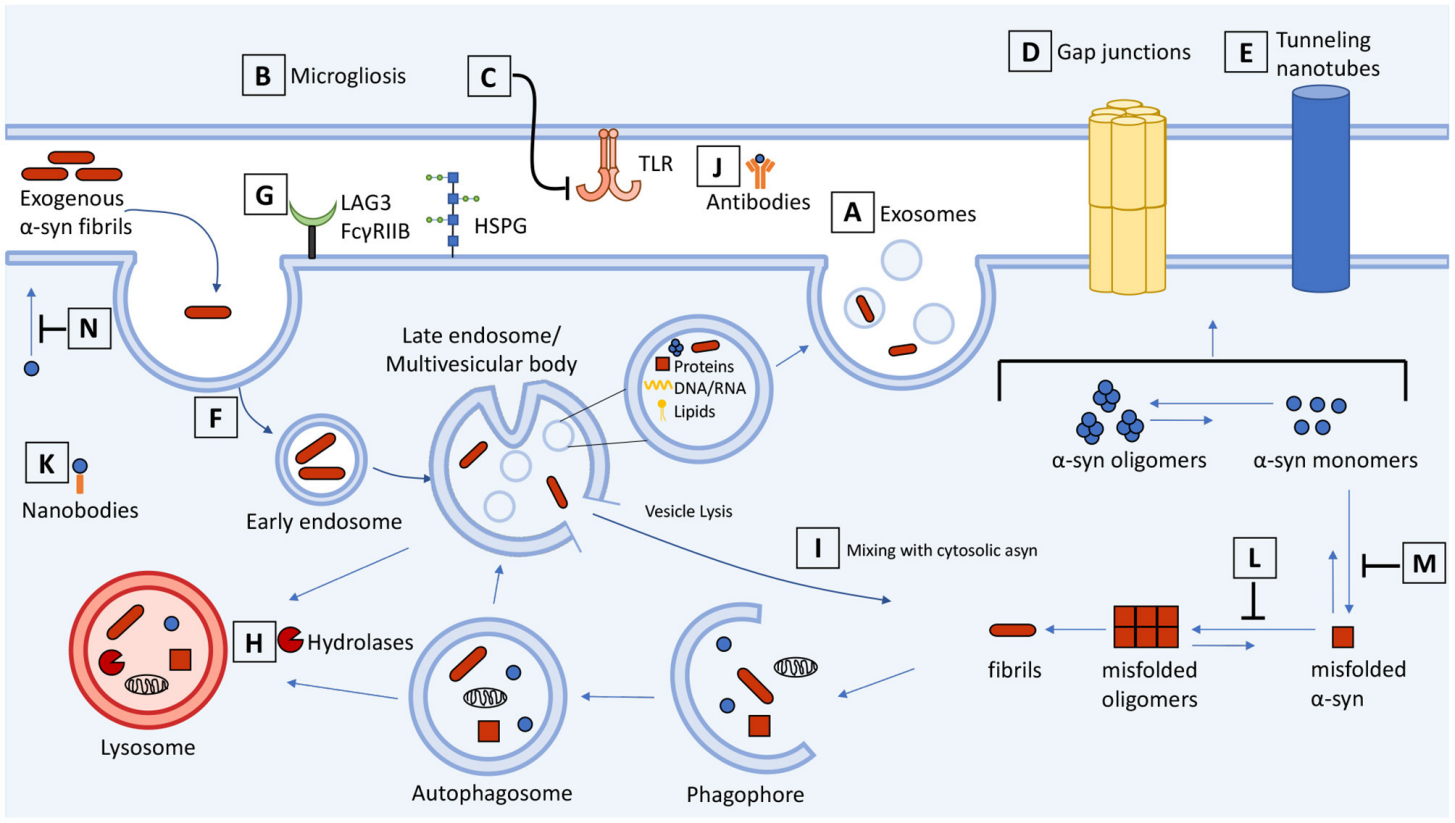
Schematic representation of cell-to-cell transmission, internalization, and trafficking of α-synuclein and therapeutic targets along the pathways. Molecular mechanisms summarized in the context of neuron-neuron and neuron-glial transmission. **(A)** Secretion and exosome-mediated release of asyn from neurons and **(B)** activated microglia contributes to the spread of asyn. **(C)** Inhibitors of microgliosis and toll-like receptors reduce pathological transmission of asyn. Transmission of asyn is also mediated through direct cell-to-cell contact through **(D)** gap junctions and **(E)** tunneling nanotubes. Internalization of asyn occurs through **(F)** endocytosis, **(G)** macropinocytosis, and receptor-mediated endocytosis which can be targeted to attenuate the transmission of asyn. **(H)** Once internalized, asyn localizes to the endosomal compartments where it is degraded by the autophagosome-lysosome pathway. Drugs that upregulate autophagy or restore lysosome function can promote the clearance of asyn and prevent its accumulation. **(I)** Internalized asyn fibrils can rupture endocytic vesicles, enter the cytosolic compartment, and induce templated misfolding of native asyn. **(J)** Antibodies can bind and sequester extracellular asyn. **(K)** Antibody fragments can be expressed inside the cell and bind to and enhance turnover of intracellular asyn. **(L,M)** Compounds can inhibit asyn from misfolding and aggregating as well as **(N)** inhibit lipid-induced asyn aggregation by displacing it from the membrane. The therapeutic targets and drugs are listed in Table 1.

An active mechanism that can enable intercellular asyn transmission is *via* small extracellular lipid bilayer vesicles known as exosomes, which contain proteins and RNA and are secreted by all cell types (Figure 1A) (42). These vesicles originate from the endosomal pathway and are generated when the late endosomal compartment known as a multivesicular body (MVB) fuses with the plasma membrane, releasing intralumenal vesicles as exosomes as well as cytosolic components (43). Post-mortem analyses of synucleinopathy brains have revealed dysfunction in the autophagy-lysosomal pathway, which is connected to the endosomal pathway and exosome release. Impairments in the clearance of autophagic vesicles contributes to the accumulation and aggregation of asyn in synucleinopathies (44). In this scheme, exosomes may represent a neuroprotective response to remove pathological asyn intermediates from cells that have defective clearance pathways. Numerous studies have demonstrated that pharmacological or genetic inhibition of the autophagy-lysosomal pathway increases the release of exosome-associated α-syn and promotes its intercellular transfer (37, 45–47). The exosome lumen also provides a concentrating milieu to facilitate further asyn self-assembly, and consequently, exosomes can transport these replicating particles to different brain areas (48, 49).

**Table 1:** Therapeutic targets and drugs from Figure 1.

**Target**	**Drugs**
1) Inhibitors of microglial activation Figure 1B	Hypoestoxide Lenalidomide Candesartan cilexetil
2) Microglial toll-like receptor inhibitors Figure 1C	NPT520-34 CU-CPT22
3) Gap junction blockers Figure 1D	CBX Gap3211 Gap2409 Gap2605
4) Endocytosis inhibitors Figure 1F	Dynasore Sertraline
5) Autophagy and lysosome inducers Figure 1H	Rapamycin Metformin Trehalose Nilotinib KYP-2407 Ambroxol AR7
6) Misfolding inhibitors Figures 1L,M	NPT200-11 Clr01 EGCG Anle1386 Plant extracts and phytochemicals SynuClean-D Apigenin
7) Lipid-induced aggregation inhibitors Figure 1N	ENT-01 Trodusquenine NPT200-11

An important side-benefit of CNS-derived exosomes, if they are indeed bona fide mediators of disease, is their potential for use as biomarkers. Analyses of plasma CNS-derived exosomes from PD patients and healthy controls reveal correlations between the amount of exosomal asyn with PD diagnoses and severity (50–52). In another study, the ratio of multimeric and phosphorylated asyn species to total asyn was higher in PD patients' plasma exosomes when compared to healthy controls (53). Together, these studies suggest that pathological exosomal cargo could serve as an early warning system for diagnosing PD.

Exosomes also play a key role in neuroinflammatory pathways that exacerbate asyn pathology. While microglia provide support in brain by phagocytosing dead cells and clearing asyn aggregates, they also release exosomes that may contribute to the spread of asyn pathology. Exposure of asyn-laden microglia to an inhibitor of exosome synthesis reduced asyn transmission to adjacent neurons and, conversely, treatment of mice with a microglial depleting compound partially suppressed asyn aggregation and transmission (54, 55). There was also less asyn aggregation in these neurons suggesting that microglial exosomes may be a therapeutic target to halt the spread of aberrant α-syn (Figure 1B). In addition, interaction of aggregated asyn with TLR2 and TLR4 on microglia induces microglial activation contributing to neurotoxicity (56–60) (Figure 1C). Microglia activated by oligomeric asyn increased their secretion of proinflammatory cytokines, and microglia-derived exosomes induced asyn pathology and apoptosis in neurons (54, 55, 61). The anti-hypertension drug candesartan cilexetil inhibited the expression of TLR2 and reversed the activated proinflammatory response of primary microglia exposed to oligomeric asyn *in vitro* (62). Modulation or inhibition of NF-κB signaling with hypoestoxide and lenalidomide, respectively, reduced microgliosis, prevented the loss of dopaminergic neurons, and reduced motor behavioral deficits in mouse models of PD (63, 64). Microglia are clearly involved in propagating asyn pathology and drugs that prevent microglial activation could be a therapeutic approach for synucleinopathies.

Transfer of multimeric asyn in neurons and oligodendrocytes can occur through direct contact with the gap junction protein connexin-32 (Cx32) (Figure 1D). Pharmacological blockade of Cx32 activity with CBX or synthetic peptide mimetics Gap3211, Gap2409, and Gap2605 decreased oligomeric asyn uptake in a concentration dependent manner (65). Likewise, asyn enhances opening of connexin-43 hemichannels in astrocytes and pannexin-1 channels, activating cytokines and inducing cell death (66). While there are conflicting findings reviewed here (67), the evidence that blocking gap junctions is beneficial in neurodegenerative models is exciting for the development of new therapeutics.

Tunneling nanotubes (TNTs) also permit asyn exchange through direct cell-to-cell contact (68–70) (Figure 1E). These membranous channels are composed of F-actin and provide plasma membrane continuity between remote cells to facilitate the transfer of vesicles, organelles and cytoplasmic molecules (71). The addition of exogenous asyn fibrils to cells induces TNT formation and fibrils are directed to the lysosome for degradation. The TNTs are a means by which cells can eliminate damaged lysosomes containing asyn fibrils, though this process can enable further asyn seeding in nearby cells (68). These TNTs are also induced as a “defense mechanism” to actively transfer toxic asyn to healthy astrocytes (70), human neuronal precursors (72), or microglia (73) which can efficiently degrade fibrillar asyn. The Wnt/Ca^2+^ pathway and βCaMKII are involved in the formation of TNTs and the transfer of vesicles and aggregated asyn (74). Drugs that destabilize TNTs or modulate the Wnt/Ca^2+^ pathway could impair TNT-mediated propagation of asyn. Although current evidence suggests that TNTs could be a target for therapeutic intervention, the contribution of TNT-mediated transfer *in vivo* and to synucleinopathies is unclear. Therefore, there is a need to assess TNTs in various cell types and animal models to distinguish contexts where TNTs contribute to asyn pathology vs. when they carry out normal physiological functions.

### Internalization of Asyn

Insights into asyn uptake are relevant to understanding the relationship between neuronal function and pathological spread, and for developing therapeutics. Extracellular aggregated asyn is cytotoxic when added to cell culture and is implicated in disease (12, 15). Internalization of asyn fibrils and oligomers occurs through temperature- and dynamin-sensitive endocytosis (75–77) along the entire length of the neuron (soma, dendrites and axons) (15, 78, 79) (Figure 1F). Once internalized, asyn moves through the endosomal compartments before being degraded by the lysosomes. This mechanism of receptor-mediated endocytosis and lysosomal degradation results in the clearance of potentially toxic asyn aggregates from the extracellular space (75). Inside the neuron internalized asyn forms inclusions in recipient cells and induces apoptosis (12, 15).

*In vivo* studies also demonstrate intercellular transfer of ectopically-expressed human asyn from rat and mouse host brains to dopaminergic neuron grafts, where the transferred asyn localized to endosomes and seeded aggregation of asyn (13, 80). Neuronal internalization of asyn was decreased when endocytosis was blocked by co-injection of the dynamin inhibitor Dynasore (13). Other mechanisms of asyn endocytosis include: macropinocytosis, an actin-dependent process that utilizes heparan sulfate proteoglycans (HSPGs) (18, 81, 82) and N-linked glycan-dependent binding to neurexin 1β mediated by N-acetylation of asyn (83), lymphocyte-activation gene 3 (LAG3) receptors (84, 85), and FcγRIIB receptors (86) (Figure 1G). There is hope that these pathways may be therapeutically targetable to attenuate cell-to-cell transmission of asyn. However, the relative contributions of each pathway to pathogenicity is poorly understood, and their cell type and species specificity remain under investigation. For example, the localization and role of LAG3 in propagating synuclein pathology has been recently questioned (87).

Filamentous asyn inclusions have also been described in glial cells of synucleinopathy patients (88). Asyn multimers may be transmitted to glial cells and further contribute to disease progression. Neuronal and astrocyte co-cultures demonstrated cell-to-cell transmission of asyn through endocytosis, forming inclusion bodies, and inducing an inflammatory response (89). These asyn multimers then localize to lysosomes in astrocytes where they can be degraded (90). Neuron-to-oligodendroglia transmission of asyn by dynamin-dependent endocytosis (91) and neuron-to-microglia transmission *via* GM1 ganglioside and lipid rafts have also been reported (92). Identification of receptors that bind asyn aggregates in neuronal and non-neuronal cells might provide alternative therapeutics that inhibit asyn internalization or promote clearance from the extracellular space to prevent asyn spread.

Post-translational modifications can also affect asyn uptake. Modification with O-linked N-acetylglucosamine (O-GlcNAc) inhibits asyn aggregation and reduces its toxicity to neurons *in vitro* (93). Inhibitors of glycoside hydrolase O-GlcNAcase (OGA) increase O-GlcNAc modification of amyloid proteins (94). Recently, OGA inhibition with a brain-penetrant inhibitor Thiamet-G increased levels of O-GlcNAc-modified proteins and impaired the uptake of asyn fibrils in mouse primary neurons (95). Pharmacological induction of O-GlcNAc could potentially be useful as a therapeutic to prevent aggregation and reduce spread of asyn.

### Intracellular Trafficking and Accumulation

Following internalization, asyn fibrils are trafficked through endosomal compartments from early to late endosomes and finally to lysosomes where they can persist for days (18, 75, 79, 84, 91). A recent study demonstrated that neutral autophagosomes and autolysosomes are located near the center of the cells at the microtubule organizing center (MTOC), and following acidification, the mature autophagosomes and autolysosomes containing asyn are transported to the periphery of the cell. In contrast, lysosomes are generated in the periphery and transported to the MTOC where they fuse with autophagosomes (Figure 1H). Thus, the lumenal pH of these vesicles is believed to regulate their subcellular localization. Asyn aggregates are located at the MTOC where autophagy occurs and once localized to vesicles they are also transported to the periphery (96). The purpose of this subcellular segregation could be to separate lysosome reformation from lysosome fusion and to secrete indigestible asyn aggregates through the plasma membrane as is the case with secretory autophagy (97).

Cells have several routes to degrade asyn, with the autophagosome-lysosome pathway bearing the primary responsibility for asyn degradation. Lysosome inhibition can accelerate intracellular inclusion formation (12, 18, 77, 98). Proteasomal inhibition has comparatively little effect, though variable results between studies could be due to differences in cell lines and inhibitors used (12, 99, 100). Chaperone-mediated autophagy (CMA) and macroautophagy can also process asyn degradation (101–103). For example, CMA-specific activator AR7 [7-Chloro-3-(4-methylphenyl)-2H-1,4-benzoxazine] increases lysosome activity and was found to attenuate asyn oligomer accumulation *in vitro* (104). Lysosomal processing and integrity are important for the clearance of asyn seeds and autophagy is a potential therapeutic target in the treatment of synucleinopathies.

Mutations in the *GBA1* gene, which encodes the lysosomal enzyme glucocerebrosidase (GCase), are a risk factor for sporadic PD and DLB (105). GCase is a lysosomal hydrolase that degrades the glycosphingolipids, glucosylceramide and glucosylsphingosine, into ceramide and sphingosine. The mechanisms by which GBA1 mutations increase the risk of synucleinopathies are not fully understood. A reciprocal association between GCase activity and asyn has been demonstrated. Elevated glucosylceramide levels due to reduced GCase activity, can lead to asyn accumulation and, conversly, increased GCase activity reduces asyn accumulation and prevents nigrostriatal degeneration (106–108). Preclinical evaluation of venglustat, a brain-penetrant inhibitor of glucosylceramide synthase, reduced asyn pathology and the associated cognitive decline in mouse models of GBA1-related synucleinopathy (109). However, a Phase 2 clinical trial (NCT02906020) testing venglustat in *GBA*-PD patients was terminated after missing its primary and secondary endpoints. Ambroxol, another drug commonly used in cough medicine, can restore GCase activity and lysosomal function and also reduce markers of oxidative stress in *GBA1* fibroblasts (110, 111). Recently, a clinical trial with 17 PD patients determined that ambroxol was safe, well tolerated, crosses the blood-brain barrier, and binds the β-glucocerebrosidase enzyme (112). While promising, future placebo-controlled trials are required to determine if ambroxol therapy could be a disease modifying intervention for PD.

Another effect of asyn aggregation is mediated through its actions at the endoplasmic reticulum (ER), which becomes fragmented in PD, impairing proteostasis and the activation of unfolded protein response (UPR), and retention of GCase within the ER (113). Interestingly, the asyn-induced deficit of ER proteostasis was rescued by inhibitors of the ryanodine receptors which modulate ER calcium influx, including with diltiazem, an FDA approved drug for hypertension and angina. Binding of asyn to the cytosolic SNARE protein ypkt6 separately interferes with ER-Golgi trafficking, which can be corrected with farnesyl transferase inhibitors (114). The combined inhibition of the ryanodine receptors and farnesyl transferase was found to correct the GCase trafficking and also lowered asyn levels in PD-derived cells, suggesting that rescue of proteostasis and trafficking in synucleinopathies may be targeted with small molecule drugs (113).

Rapamycin, an FDA approved drug, increases autophagy levels by inhibiting the mTOR pathway and can reduce dopaminergic cell death, decrease levels of phosphorylated asyn, and improve mitochondrial function in cell and animal models of synucleinopathy (115–117). Rapamycin also interferes with other autophagy independent pathways and therefore, the downstream target of mTOR, lysosomal transcription factor EB (TFEB), may be a more appropriate target. TFEB overexpression decreased pathologic asyn oligomers in rats (118), and TFEB overexpression in oligodendroglia rescued nigrostriatal neurodegeneration in an MSA mouse model (119). In contrast to rapamycin, metformin, trehalose, and nilotinib induce AMP-activated protein kinase (AMPK)-dependent autophagy. This can also prevent asyn accumulation by promoting its clearance and is neuroprotective in PD models (120–122). To determine the synergistic effects of mTOR-dependent and -independent autophagy stimulation, trehalose and rapamycin were tested in an MPTP-induced PD mouse model and showed an additive effect in recovery of dopaminergic neurons and neuroprotection (123). However, one concern of inducing autophagy is the potential co-clearance of other proteins with differential turnover rates (124). While upregulation of autophagy is generally beneficial (125, 126), high autophagic flux can also induce cell death (127). Understanding the precise pathways of asyn clearance and homeostasis will be important for the development of safe autophagy-based therapies.

Direct contact between misfolded and native asyn is necessary for permissive templating. How is this interaction mediated if misfolded asyn is lumenal, enclosed within endocytic vesicles, whereas the native asyn substrate is cytosolic? There is evidence that asyn aggregates can rupture endocytic vesicles following internalization (76, 128, 129) (Figure 1I). The ruptured vesicles can also fuse together to form LB inclusions, which are heterogenous in their composition, containing a mixture of asyn filaments, vesicle lipids and fragmented membranes, and other organelles (130, 131). To prevent these pathological structures, the porphyrin phtalocyanine tetrasulfonate was used to bind to and stabilize vesicle-associated asyn to block its misfolding and aggregation (132). Despite these advances, our knowledge of the molecular players and mechanisms involved in the internalization and transmission of asyn remains inadequate and poses a challenge for identifying therapeutics that target asyn transport.

## Therapeutics Targeting Asyn Levels

### Targeting Extracellular Asyn

The ability of antibodies to sequester misfolded proteins with high-affinity and high-specificity make them attractive as therapeutics for neurodegenerative diseases (Figure 1J). Extensive research over the past two decades has focused on developing active and passive immunity to target extracellular asyn and several anti-asyn vaccines are currently being tested in early phase clinical trials (133). The initial vaccine studies from Masliah and colleagues were encouraging and showed that both active and passive immunization could lower intracellular asyn aggregates and rescue motor dysfunction in transgenic asyn overexpressing mice (134, 135). Subsequent work has targeted carboxyl-terminal asyn epitopes. Reports of active immunization indicate improved immune cell recruitment and high antibody titers that selectively reduce asyn pathology (136, 137). Antibodies generated to asyn oligomeric species recognize asyn aggregates in human PD tissue but were not sufficient to rescue pathology or the motor phenotype in transgenic mice overexpressing human asyn (138). Whether this was due to specificity of the immunizing oligomer conformation or disease vs. overexpression differences is unclear. Active immunization also carries the risk of generating an autoimmune response against asyn, which can be minimized by passive immunization using humanized or engineered antibodies. Preclinical testing of such antibodies showed promise in preventing neurodegeneration and rescuing motor deficits in mouse and primate models of synucleinopathy (135, 139–145). These findings prompted the testing of a humanized IgG1 monoclonal antibody PRX002 (Prasinezumab, Roche/Prothena) that recently completed its Phase 2a clinical trial. Although the study could not meet its primary outcome measure of change in patient MDS-UPDRS score from baseline, some motor scores showed positive trends, and the study has now progressed to Phase 2b of the trial (NCT03100149)(146).

Active immunization with peptides mimicking the asyn carboxyl-terminus effectively reduced pathological aggregate formation and significantly mitigated motor deficits in transgenic human asyn mice (147). These peptides were further developed by Affiris as PD01A and PD03A (AFFITOPE) for active immunization clinical studies. Importantly, Phase I clinical trials using these antibodies in patients with PD were successfully completed in 2016, showing that both antibodies were locally and systemically well tolerated with no serious adverse effects, which resulted in their progress into Phase II clinical trials (148–150). In addition, a synthetic asyn peptide representing oligomeric and fibrillar asyn developed by United Neuroscience (UB312) is currently undergoing a Phase 1 clinical trial for PD (NCT04075318) (151).

Biogen's BIIB054 (Cinpanemab) monoclonal antibody, on the other hand, did not meet its primary and secondary endpoints in a Phase 2 clinical trial, despite promising pre-clinical results (152), and the trial was discontinued in February 2021 (NCT03318523). Several other antibodies targeting asyn are currently in Phase I trials for passive immunotherapy, including MEDI1341 (AstraZeneca), Lu-AF82422 (Lundbeck), ABBV-0805 (AbbVie/BioArctic), ABL301 (Sanofi/ABL Bio) and UCB7853 (UCB Biopharma/Novartis) (clinicaltrials.gov).

Notwithstanding the significant advances in immunotherapy as a disease modifying strategy, achieving adequate BBB penetrance and target engagement remain key challenges for the use of these therapeutics in the clinic. Retrospective evaluations of multiple immunotherapy clinical trials conducted for Alzheimer's disease (AD) have shown limited benefits (153, 154). The low immunoglobulin levels detected in CSF (<0.5% of serum IgG levels), and lower still in the brain parenchyma, may account for the absence of therapeutic benefits (155). Strategies to improve BBB passage are therefore an important and urgent requirement for developing efficient therapeutic antibodies. Another potential challenge remains the structurally distinct asyn strains that have been identified in human synucleinopathy brains (156–159). These conformers represent reproducibly stable and distinct conformations of asyn. It is unclear whether these be preferentially targeted by conformation-specific immune approaches. The possibility that immune reagents could be developed to target pathogenic conformations of asyn is very appealing, particularly if the non-disease asyn conformers were selectively spared.

### Reduction of Intracellular Expression of the Asyn Gene SNCA

As a complementary strategy to targeting extracellular asyn, reducing expression of SNCA (the gene encoding asyn) and intracellular asyn levels in recipient neurons could also mitigate disease progression by reducing the native substrate available for templated misfolding. RNA interference (RNAi) has been widely used in cell and animal models to effectively counteract asyn accumulation. Small interfering RNA (siRNA) are 21–23 nucleotide long double stranded RNA molecules that drive degradation of target mRNA upon sequence-specific binding. Direct infusion into mouse hippocampus of naked anti-asyn siRNA successfully downregulated SNCA expression (160). Short hairpin RNA (shRNA), which is processed by the cellular machinery to form active siRNA, has also been used to lower SNCA expression in neuronal cultures (161), in murine models (162–164), and in non-human primates (165). Although these studies have shown that downregulation of asyn expression significantly reduces asyn transmission, reducing asyn expression *in vivo* has potential disadvantages including possible neuronal dysfunction, neurotoxicity, and loss of dopaminergic neurons. *In vivo* studies on the viability of this approach for counteracting PD have been controversial; some reports suggest that >90% suppression of asyn could be neurotoxic and cause the loss of nigral tyrosine hydroxylase expression (165–167), whereas other studies have not observed toxicity with up to 60–70% asyn knockdown (164, 168). Cumulatively, the evidence suggests that partial knockdown of asyn levels is safely achievable, and that even a moderate 30–40% asyn reduction is beneficial against spreading asyn pathology (169, 170). However, if asyn knockdown is found to induce some loss-of-function deficits, it is conceivable that these might be circumvented by corresponding expression of a non-aggregation prone homolog, such as β-synuclein (bsyn), which lacks the hydrophobic core of the non-amyloid component (NAC) domain.

Genome-wide association analyses (GWAS) have revealed that expression profiles of certain micro RNAs (miRNAs) including include miRNA-30, miRNA- 485, miRNA-29, miRNA-26, and let-7 are altered in the brains of PD patients (171, 172). MicroRNAs are small non-coding RNAs that post-transcriptionally regulate target mRNAs and their therapeutic potential in the treatment of PD has consequently generated considerable interest. Four different miRNAs including miR-7, miR-153, miR34b/c, and miR-214 were found to directly regulate asyn, suggesting a potential role for these miRNAs in asyn accumulation and disease progression (173–176). Moreover, incorporating an asyn targeting sequence within an miR-30 structure was shown to improve SNCA silencing and provide protection against dopaminergic neuronal loss and motor deficits in a murine model of PD that overexpresses human asyn (177). AAV9-mediated expression of miR-30-SNCA-shRNA in mouse brain also suppressed asyn levels (168). Moreover, AAV1 expression of an artificial miRNA bearing an asyn targeting sequence reduced the spread of asyn pathology and the associated motor deficits in an aggressive mouse model induced by intracerebral inoculation with synucleinopathy brain homogenates (170).

Delivery vectors for nucleic acid-based therapies can be broadly classified into non-viral and viral vectors. Non-viral vectors display low cytotoxicity and immunogenicity, are cost-effective, and can be easily modified for target binding (178). For example, non-viral vectors for applications in PD include modified peptides, polymers, lipids, exosomes and inorganic materials, or hybrid systems comprising combinations of these vectors. A key disadvantage of non-viral therapeutics is their transient action and requirement for periodic re-administration. Transient asyn suppression can be induced with anti-sense oligonucleotides (ASOs), which are single-stranded oligonucleotide sequences that activate endonuclease RNaseH upon binding to the target mRNA and result in the degradation of the DNA-RNA hybrid. Studies employing asyn ASOs have reported reduced asyn protein expression without causing dopaminergic neurodegeneration (169, 179, 180). BIIB101, an anti-asyn ASO developed by Ionis and Biogen is currently in a Phase I clinical trial in MSA patients (NCT04165486). Results from this study, if successful, will undoubtedly pave the way for ASO-based therapeutics for PD. Despite their relatively long half-life, ASOs will require regular intrathecal administration to maintain stable asyn silencing.

Viral vectors have been historically preferred for gene therapy targeting neurodegenerative disorders because of their stable and long-term expression profiles. Adeno-associated viral vectors (AAVs) are currently the most advanced vectors for CNS gene therapy, with AAV serotypes 1,2,5 and 9 being the most frequently used (34, 181). Lentiviral vectors have also been tested for PD therapeutics, as they allow larger DNA inserts and can encode multiple genes simultaneously. Lentivirus-based PD clinical trials have focused primarily on augmenting dopamine levels by expressing multiple enzymes necessary for its synthesis (182). Viral vectors do have some disadvantages, including inherent immunogenicity, species-specific tissue-tropism and challenges with cost-effective manufacture of virion particles, which may restrict their widespread application (183).

Direct gene editing is being developed as an interesting alternative to repeated administration. Although it is still at an early preclinical stage, gene editing bears the potential to fundamentally alter how genetic disorders are treated by enabling fine tuning of disease gene expression or by correcting mutations. For example, zinc-finger nuclease (ZFN) mediated genome editing was used to correct point mutations in the *SNCA* gene in patient-derived human induced pluripotent stem cell (hiPSCs) (184). Gene editing with the CRISPR/Cas9 system targeted a DNA methylation site to successfully reduce asyn expression in human iPSC-derived neurons from a PD patient with the SNCA triplication (185). Recently, a CRISPR-based approach was used in transgenic AD-related animals to selectively target the mutated amyloid precursor protein gene, while leaving wild-type alleles intact (186). This significantly reduced APP amyloidosis and gliosis. Although much research is required to explore its full potential, genome editing has immense potential for the precision treatment of genetic disorders such as PD.

A central focus of CNS targeted gene therapy is the development of vectors that can efficiently bypass the blood brain barrier (BBB). The BBB is highly selective and restricts the entry of molecules larger than 400 daltons such that it is impermeable to the vast majority of systemically administered small molecule drugs and nearly all biological therapeutics (187) (discussed further in Section Non-invasive Drug Delivery to Brain). Although direct intracerebral injections are commonly used for gene transfer in pre-clinical models, they are impractical for CNS disorders that require broad bilateral gene expression. Invasive needle tracts induce mechanical trauma to the surrounding tissue, exacerbate local neuroinflammatory processes, and increase the risk of hemorrhage. Intranasal delivery is another potential delivery route to circumvent the BBB (179). It is non-invasive and practically feasible, but low dosage volume, susceptibility to degradation by mucosal enzymes, and substantial variabilities in target engagement make this delivery option suboptimal at present (188). Hence, vectors that can efficiently transfer therapeutic payloads across the BBB are important for the success of gene therapy approaches. Recently, novel brain-penetrating AAV9 variants, such as AAV-PHP.B, have been generated by selecting among random modifications of the viral capsid (189–192). Preclinical testing of these novel AAVs have shown efficacy in ameliorating rodent disease models following systemic delivery, but challenges with differential species- and strain-specific tropism remain (193). The gene therapy vectors that have been used to knockdown *SNCA* in various PD models have been summarized in Table 2.

**Table 2:** Delivery vectors for asyn targeted gene therapy.

**Vector type**	**Vector**	**Oligonucleotide**	**References**
Viral	AAV	*SNCA*-shRNA	(162, 164, 166–168, 177)
		*SNCA*-miRNA	(163)
		*SNCA*-ribozyme	(194)
	Lentiviral	*SNCA*-shRNA	(161)
Non-Viral	Peptides	*SNCA*-siRNA	(195, 196)
	Gold nanoparticles	*SNCA*-siRNA	(197)
	Polyethylenimine nanoparticles	*SNCA*-siRNA	(198)
	Exosomes	*SNCA*-siRNA	(199)
		*SNCA*-shRNA-minicircles	(200)
		*SNCA*-ASO	(180)
	Indatraline	*SNCA*-siRNA	(201)
		*SNCA*-ASO	(179)

### Reduction of Intracellular Asyn Protein Expression Using Gene Therapy

The primary drawback of using antibodies is their high molecular weight, which challenges their therapeutic potential. Intrabodies (also called nanobodies) are small antibody fragments containing only the antigen-binding domain, derived from either heavy-chain only antibodies (HCAbs) found in all Camelidae and some shark sera or recombinantly produced from conventional antibodies (202). The two main types of nanobodies are short chain variable fragments (scFv), which consists of the variable light and variable heavy chains linked with a polypeptide sequence, and single-domain (sd) nanobodies (Figure 1K). Among the advantages that nanobodies have over conventional antibodies is their smaller size (typically 15–30 kDa), allowing for greater tissue penetrance, and faster clearance (203–205). Their smaller size (scFv: 250 aa, sdAbs: 120–140 aa) also enables their recombinant DNA to be easily vectorized for gene delivery (206). Given that only a small proportion of peripherally administered antibodies reach the CNS, vectorized intrabodies may overcome this challenge by enabling expression in CNS neurons. The lower immunogenicity, and ability to genetically engineer fusion sequences also enhance the safety and functional potential of intrabodies.

Multiple scFvs targeting different conformations and epitopes of asyn have been selected either using phage display alone or in combination with atomic force microscopy, previously reviewed (207, 208). A recent study produced a scFv from a monoclonal antibody, specific for asyn fibrils (Syn-F2) and found it was able to inhibit seeding of asyn aggregation *in vitro*. This intrabody was fused with a cell-penetrating peptide, underscoring the ability for genetic engineering to enhance the functionality of intrabodies (209). Despite the ability of anti-asyn scFvs to inhibit formation of toxic oligomeric asyn *in vitro*, only a few have been further tested in synucleinopathy models to date. NbSyn87 is a camelid-derived nanobody, selected using phage-display with affinity for epitopes in the N-terminal and C-terminal of asyn, allowing it to bind to both monomeric and early fibrillary forms of asyn (210). NbSyn87 inhibited the formation of toxic oligomers *in vitro* (211) and, following expression in an asyn-overexpression rodent model, efficiently cleared phosphorylated asyn (p-asyn) and partially rescued motor dysfunction (212). The same study also tested a single-domain nanobody, VH14, a human-derived clone with high affinity for the NAC domain of asyn. While both nanobodies were effective, VH14 produced a greater reduction of p-asyn deposits, and unlike NbSyn87, did not generate an inflammatory response (212). One shortcoming of intrabodies is their relatively poor solubility in the cytoplasm, though this can be improved with further engineering. For example, fusion of a protein degradation sequence rich in proline, glutamine, serine and threonine, hence the name PEST, was shown to improve the intracellular solubility of various anti-asyn nanobodies including VH14 and NbSyn87 *in vitro* by increasing their overall negative charge (213). PEST also functions as a ubiquitin-independent proteasome-degradation signal, thereby enhancing the turnover of cytoplasmic nanobody-asyn conjugates (213, 214). Engineering intrabodies to possess proteolysis targeting sequences represent a promising approach to reducing intracellular asyn concentration, thereby not only inhibiting the misfolding, but also decreasing the amount of substrate to be recruited for aggregation.

### Pharmacological Reduction of Asyn Expression and Aggregation

Targeting “druggable” cellular pathways known to modulate asyn transcription may be a useful approach to decreasing asyn levels. Table 3 summarizes pharmacological therapeutics that reduce asyn. For example, agonists of the Beta2 adrenoreceptor (β2AR) downregulate transcription of SNCA gene by decreasing histone H3 lysine 27 acetylation of its promoters and enhancers (215). Three β2AR agonists, metaporterenol, clenbuterol, and salbutamol were found to reduce endogenous SNCA mRNA in a dose- and time-dependent manner following treatment in neuroblastoma and rat primary cortical neurons. Conversely, treatment with β2AR antagonist, propranolol, increased asyn mRNA. Clenbuterol and salmeterol, both BBB-permeable, can protect dopaminergic neurons from MPTP toxicity in a PD mouse model (215, 216). Clenbuterol also significantly reduced asyn mRNA in SNCA triplication iPSC derived neuronal cells. These pre-clinical findings were strengthened by epidemiological data of Norwegian populations prescribed salbutamol (most commonly to treat asthma) and propranolol (hypertension), and salbutamol was associated with reduced risk of developing PD (rate ratio of 0.66) and, conversely, propranolol was associated with increased risk of PD (rate ratio 2.20). Additional epidemiological investigations have reported the same relationship (240) or no association (241). In addition to its SNCA transcription regulation, β2AR agonists also have anti-inflammatory effects (216), making it an especially promising therapeutic target in PD. Although β2AR agonists have shown to be safe and effective in other neurological conditions including multiple sclerosis (242) and ALS (243), no large scale clinical trials in PD have been conducted to date. However, three open label trials observed that salbutamol added to levodopa treatment provided some symptomatic benefit in a small number of PD patients tested (244–246).

**Table 3:** Pharmacological approaches to reduce asyn.

**Small molecule**	**Origin**	**Mechanism of action**	**Model(s)**	**Clinical stage**	**References**
*Beta2 adreno receptor agonists* **1. Metaporterenol** **2. Clenbuterol** **3. Salbutamol**	Synthetic	Agonists decrease transcription of SNCA gene.	Primary rat neurons; neuron precursors derived from iPSCs from patient with SNCA-triplication. MPTP mice: protected TH+ neurons. WT mice: reduced expression of asyn in SN.	Synucleinopathies: N/A	(215)
**4. Salmeterol**	Synthetic	Inhibits transcription of pro-inflammatory cytokines	MPTP mice: protected DA neurons against induced toxicity.	N/A	(216)
*Disaggregation of asyn*					
**1. NPT200-11 (UCB0599)**	Synthetic	Inhibits lipid-dependent nucleation	PDNG78 mice: reduced retinal α-syn pathology. Line 6 mouse model: reduced cortical α-syn pathology and astrogliosis, normalized striatal dopamine transporter levels, and improved motor phenotype.	Phase 2 (NCT04658186) in PD patients	(217, 218)
**2. Anle138b**	Synthetic	Anti-oligomeric	MPTP mice: preserved dopaminergic nigral neurons. Thy1-A30P mice: decreased pathological α-syn oligomers. M12 transgenic mice: reduced density of α-syn aggregates, improved dopamine neuron function and gait. MSA mice: rescued motor dysfunction, reduced α-syn oligomers and glial cytoplasmic inclusions, preserved DA neurons.	Phase Ib (NCT04685265) in PD patients	[(219–221)
**3. ENT-01 (squalamine)**	Natural	Inhibits lipid-induced asyn aggregation	SHSY5Y cells: reduced asyn oligomer-induced toxicity. *C.elegans* overexpressing asyn: restored motor dysfunction.	Phase 2b KARMET (NCT03781791) in PD patients. Phase I (NCT03938922) PD dementia	(222)
**4. Trodusquemine (MSI-1436)**	Natural	Inhibits lipid-induced and fibril-induced asyn aggregation	SHYSY cells: reduced oligomer-induced toxicity. *C.elegans* PD model: reduced asyn inclusions, increased longevity.	N/A	(223)
**5. CLR01**	Synthetic	Molecular tweezer inhibits asyn aggregation through binding to lysine residues of asyn critical for its oligomerization	Zebra fish-asyn: improved phenotype, and survival, suppressed α-syn aggregation. Thy1-asyn mice: attenuated motor dysfunction, however, did not reduce aggregated α-syn levels. LB treated iPSCs: decreased aggregation and toxicity. LB and pff-seeded mice: reduced asyn pathology and DA neuron rescue.	N/A	(224–226)
**6. EGCG**	Natural	Inhibits asyn fibrillogenesis, converts fibrils into amorphous, non-toxic protein aggregates.	HEK-293 WT asyn; MPTP mice: rescued motor dysfunction, protected TH+ SN neurons, increased dopamine expression, reduced α-syn. MPTP-monkeys: reduced oligomeric α-syn in striatum, increased nigral TH+ neurons and DA levels, improved motor dysfunction.	Phase 3 completed. PROMESA clinical trial of EGCG in patients with MSA, failed to show a protective effect.	(227–229)
**7. Phenylbutyrate**	Natural	Histone deacetylase inhibitor, increases DJ-1 production levels.	MPTP mice: protected dopaminergic neurons and preserved motor and cognitive functions. Increased brain α-syn clearance, reduced neuroinflammation.	Completed Phase I (NCT02046434) in PD patients.	(230, 231)
**8. SynuClean-D**	Synthetic	Binds to fibrils and disrupts their nucleation and elongation.	*C.elegans* PD models: decreased asyn aggregation, rescued dopaminergic neurodegeneration, and restored motor function.	N/A	(232)
**9. Apigenin**	Natural	Slows secondary nucleation of asyn.	Cell free assay: reduced number of oligomers formed in asyn aggregation assay.	N/A	(233)
*Modulating autophagy* **10. KYP-2047**	Synthetic	Prolyl oligopeptidase inhibitor	Asyn pff-SHSY5Y cells: increased degradation of HMW asyn, reduced α-syn monomers and α-syn secretion. Cell lines over-expressing mutant α-syn: reduced α-synuclein inclusions, improved cell viability. A30P-asyn mice: reduced soluble α-syn, improved motor symptoms	N/A	(234–236)
**11. NPT520-34**	Synthetic	Toll-like receptor 2 antagonist. Facilitates clearance of misfolded asyn *via* upregulating autophagy	L61 mice: reduced accumulation of α-syn and neuroinflammation, preserved dopamine signaling in brain, improved motor function.	Phase 1b	(172, 237)
*Iron related molecules*					
**12. ATH434**	Synthetic	Redistributes labile iron	MPTP, 6-OHDA, A53T α-syn mice: protected SNpc neurons, reduced asyn accumulation, improved motor performance. MSA mice: preserved dopaminergic nigral neurons, reduced asyn oligomerization.	Completed Phase I in healthy elder volunteers. Plans for Phase 2.	(238)
**13. Synucleozoid**	Synthetic	Downregulates SNCA mRNA translation	SHSY5Y cells: reduced asyn pff-induced toxicity, decreased asyn expression.	N/A	(239)

Small molecules targeting asyn mRNA to downregulate its expression are being evaluated. A sequence-based design was used to identify molecules that bound to the iron-responsive element (IRE) of SNCA mRNA, a region involved in regulating translation (239). The most promising candidate, synucleozoid, was most effective at inhibiting the translation of asyn, and showed a dose-dependent reduction in asyn fibril-induced cytotoxicity. Mechanistically, synucleozoid lowered asyn protein levels by reducing the amount of SNCA loaded onto polysomes and, importantly, was selective for asyn mRNA (239). This study provides encouraging evidence that pharmacologically modifying the structural elements in asyn mRNA can reduce asyn expression.

Preventing asyn aggregation with small molecule inhibitors is being investigated (Figure 1L), but few candidates have reached human clinical trials. Epigallocatechin-3-gallate (EGCG), a polyphenol, was shown to prevent the conversion of asyn into toxic oligomers (227, 228) and was tested in a Phase III trial (PROMESA, NCT02008721) in patients with MSA although it failed to show efficacy, perhaps due to its low bioavailability and sub-acute toxicity in some patients (247). The lack of specificity for asyn hinders EGCG's viability as it inhibits fibrillation of at least 14 different proteins; however, it does appear to have anti-inflammatory and neuroprotective effects in preclinical and clinical trials (229). Plant extracts and phytochemicals such as isorhynchophylline, paeoniflorin, baicalein, curcumin, cuminaldehyde, gallic acid, ginsenosides, and resveratrol have been proposed as possible drug candidates for PD as they reduce asyn oligomerization and fibril formation (Figure 1M) *in vitro* and *in vivo*, reviewed by (248). Recently a small molecule kinetic-based screen was developed to identify inhibitors of asyn aggregation and the compound apigenin significantly reduced the formation of oligomers (233). Another small molecule, SynuClean-D, was able to not only reduce asyn aggregation but also rescue dopaminergic neuron degeneration in worms (232). A promising anti-aggregant from Neuropore Therapies, UCB0599, recently entered a Phase 2 clinical trial (NCT04658186) in 450 early-stage PD patients with an expected completion date in mid-2024. This second-generation compound with improved brain penetration, previously named NPT-200-11, interacts with the asyn carboxyl-terminal domain and reduces asyn pathology, inflammation, and associated motor deficits in multiple mouse models with asyn aggregation (217, 218).

As phospholipid binding may affect the formation of asyn fibrils (249), modulating asyn lipid binding could effectively reduce its aggregation (Figure 1N). Various molecules which compete with asyn for lipid membrane binding have been tested *in vitro*, although only one has advanced beyond Phase I clinical trials. ENT-01 was demonstrated to compete with asyn for membrane binding and effectively restored peristalsis in mouse models of PD (222). A 50-patient open label Phase 2a study found that orally administered ENT-01 tablets were safe and reduced constipation in over 80% of PD patients, and mildly improved both motor and non-motor symptoms, and a Phase 2b trial is ongoing assessing both constipation and neurological symptoms in 152 PD patients (KARMET, NCT03781791). Although targeting peripheral asyn within the enteric nervous system before it potentially travels to the CNS/brainstem may be beneficial, ENT-01 cannot cross the BBB. A structurally similar BBB-permeable molecule, trodusquemine, has also demonstrated ability to displace asyn and its oligomers from the membrane, inhibiting its lipid-induced aggregation *in vitro* and in a C. elegans model of PD (223), and is in development for clinical trials.

Anle138b, another BBB-permeable drug, developed by MODAG is proposed to directly inhibit inter-peptide interactions and formation of beta-sheet rich asyn structures (219). Oral administration of anle138b reduced asyn oligomers, and restored striatal dopamine and motor function in both MSA (221) and PD (220) mouse models. Anle138b treatment increased the number of small, monomeric asyn species, while decreasing the inner density of the larger insoluble asyn aggregates, suggesting it interfered with the asyn aggregation process and potentially destabilized existing asyn aggregates (220). Assessment of the tolerability and pharmacodynamics of anle138b is currently underway in a double-blind placebo controlled ascending dose trial in PD patients, with expected completion in June 2022 (NCT04685265).

## Artificial Intelligence Approaches for Drug Repurposing

Drug repurposing represents a fast and cost-effective option for developing new treatments for PD [recently reviewed in (250)]. This approach takes advantage of previously approved drugs and re-targets them for a new indication. Repurposing regulatory agency-approved molecules, with an existing safety and pharmacokinetic record in humans, bypasses the high-risk phase of the drug development process and can enter development for a new indication at Phase IIa, providing significant savings in both cost and time. Indeed, drug repurposing can cost as little as $250,000, take as little as 4 years and has a significantly greater chance of reaching patients than a novel drug (251, 252).

A recent review of all clinical trials for PD found that 50 of 145 drugs in phase I-III clinical trial for PD were repurposed, illustrating the strong contribution of repurposing as a strategy to enhance the drug development pipeline for PD (253). Notably, of these 50 repurposed drugs, only two purportedly target asyn; mannitol and memantine. Mannitol is a sugar approved for reducing intracranial pressure, cerebral edema and intraocular pressure. It can also function as a chaperone to stabilize asyn structure *in vitro* and attenuate synucleinopathy in asyn transgenic mice (254) and is currently being tested in a Phase II clinical trial for effects in PD (NCT03823638). The NMDA receptor antagonist memantine, indicated for moderate and severe AD, is currently being tested in 50 patients in a Phase II clinical trial for effects on the cell-to-cell transmission of asyn (NCT03858270). Since the review of McFarthing and colleagues, the histone deactetylase inhibitor phenylbutyrate, currently indicated for rare pediatric metabolic disorders, has also begun a small Phase 1 study to test the effects on elimination of asyn from the brain to the blood in individuals with PD (NCT02046434), having been previously shown to increase DJ-1 expression and attenuate asyn aggregation in Y39C asyn transgenic mice (230).

Despite the significant advantages over traditional drug development, a major challenge in repurposing remains the efficient identification of drugs with the appropriate efficacy and prioritizing them for development, from the thousands of regulatory approved compounds. To address this challenge, a machine learning approach using artificial intelligence was recently deployed to identify existing drugs that might prevent oligomerization of asyn. The computational approach uses text mining of published abstracts to generate mathematical vectors that can be used to rank a set of candidate drugs based on semantic similarity to compounds with a known effect (prevention of oligomerization of asyn) (255). Based on their predicted ability to inhibit the asyn aggregation, 620 approved drugs were ranked with this methodology. Pharmacoepidemiologic analysis of 15 of the top 50 drugs replicated expected associations between allopurinol and fenofibrate and PD and further identified 3 novel drugs associated with a decreased odds of incident PD; pentoxifylline, theophylline and dexamethasone (256). From a list of 1832 FDA approved drugs, several antihypertensive drugs were identified that might reduce asyn oligomerization. Combinations of angiotensin receptor II blockers and dihydropyridine calcium channel blockers, angiotensin converting enzyme inhibitors and diuretics were found to be inversely associated with time to PD diagnosis and thus be of interest for having disease modifying potential in PD (257). Recently, following an AI-based screen, several compounds were systematically evaluated in cell and animal models of asyn overexpression and revealed rifabutin as a potential drug to reduce nigrostriatal degeneration (258).

Although these data require replication in independent cohorts and/or investigation in laboratory-based assays or animal models of asyn aggregation, they do provide promising real-world data on the potential of using a computational approach to efficiently identify drugs targeting oligomerization of asyn, that are amenable to repurposing for disease modification in PD. To our knowledge there are still no drugs with regulatory approval that were identified using artificial intelligence, however given the pace of discovery and significant investment in this domain, the true promise of this technology should become apparent in the coming years.

## Non-invasive Drug Delivery To Brain

A key challenge for asyn therapeutics is their delivery across the BBB, a protective and selectively permeable microvasculature around the CNS composed of tight junctions, endothelial cells, pericytes and astrocytic end foot processes. Receptors, ion channels and transporters on the BBB tightly regulate the transport of molecules into the brain parenchyma, thereby restricting the bioavailability of CNS targeted drugs (187). Indeed, several therapeutics that have shown promise in pre-clinical studies have been unsuccessful in clinical trials, primarily because of their difficulty in crossing the BBB and poor target engagement (259).

In the past two decades, the use of MRI-guided focused ultrasound (MRIgFUS) has developed as a revolutionary technology that allows for localized and targeted delivery of CNS therapeutics across the BBB without the need for invasive surgery. Although the first demonstrated use of focused ultrasound waves resulted in BBB opening around a lesion site (260), a subsequent landmark study by Hynynen and colleagues combined intravenously injected preformed gas microbubbles with low intensity focused ultrasound (FUS) waves to safely open the BBB in rabbits (261). The coincident application of low-intensity focused ultrasound waves to the target brain areas causes the circulating microbubbles to oscillate and mechanically induce a localized and transient opening of the microvasculature tight junctions. With appropriate calibration, the inflammatory responses, thermal changes, and brain hemorrhage observed with high-intensity ultrasound can be avoided. Importantly, coupling intravenous drug delivery with the transient increase in BBB permeability in specific brain regions has been successfully used to increase the CNS bioavailability of an array of biologics including antibodies, viral vectors and nucleic acids for gene therapy in multiple species from rat to non-human primates (259, 262).

Importantly, BBB opening induced by FUS can be widely adapted to small, large, and multiple brain regions by sonicating single or multiple foci. For example, discrete or multiple foci enabled restricted or broad whole-hemisphere gene transfer and drug delivery in rodents (168, 263, 264). In humans, although the technology is still in clinical trials, brain volumes from 63 mm^3^ to 25 cm^3^ have been safely targeted with MRIgFUS for increased BBB permeability (265–269).

Several studies have evaluated MRIgFUS in preclinical models of PD. For example, MRIgFUS was used with intravenous AAV9-SNCA-shRNA to target PD-relevant brain regions as defined by Braak staging in a transgenic mouse model overexpressing human asyn resulting in a 40–60% reduction in asyn levels in the targeted brain regions (168). Another study used FUS to deliver glial cell line-derived neurotrophic factor (GDNF) in 6-hydroxydopamine (6-OHDA) treated rats and rescued dopaminergic neurons and the corresponding motor deficits (270). Liposome-mediated gene delivery of GDNF in combination with FUS was also shown to be neuroprotective in a 1-methyl-4-phenyl-1,2,3,6-tetrahydropyridine (MPTP) mouse model of PD (271). Unilateral FUS-mediated opening of mouse BBB coupled with intranasal brain-derived neurotrophic factor (BDNF) significantly enhanced BDNF uptake into the ipsilateral hemisphere of mice (272). Similarly, intranasal delivery of BDNF followed by FUS in MPTP-treated mice rescued dopaminergic neurons and behavioral deficits (273). Recent studies also seem to indicate that FUS mediated opening of the BBB by itself may promote neuroplasticity. For example, treatment with MRIgFUS alone in an AD animal model reduced tau and Aβ protein aggregates (274). The mechanism is not fully understood, but the treatment also increased neurogenesis in the dentate gyrus, possibly by transient upregulation of neurotrophic and pro-inflammatory factors. Whether FUS-mediated BBB opening induces a similar clearance of asyn aggregates remains to be determined, though neuroinflammation is associated with the accumulation of misfolded asyn (275).

With the abundant pre-clinical evidence supporting multiple applications of FUS-mediated BBB opening, its safety and feasibility in humans is now under evaluation for several neurodegenerative diseases. A Phase I clinical trial used the transcranial ExAblate system to repeatedly open the BBB in five patients with AD (NCT02343991) (266). Importantly, the integrity of the BBB was restored within 24 h in all 5 patients. A follow-up Phase IIa trial will assess the safety of targeting multiple brain regions in a larger AD cohort (NCT03739905). Another Phase I study reported the safe and reversible BBB opening in four patients with amyotrophic lateral sclerosis (ALS) (265). An ongoing clinical study (NCT03608553) is also evaluating the safety and feasibility of opening the BBB in patients with mild to moderate Parkinson's disease with dementia (268). These studies represent an important step toward the non-invasive delivery of disease modifying therapeutics for CNS diseases.

Taken together, growing pre-clinical and clinical evidence indicates that FUS mediated BBB opening offers several advantages for therapeutic delivery. First, the FUS mediated increase in BBB permeability is non-invasive and therefore, repeated therapeutic applications, if required, may be a viable strategy. Second, MRIgFUS allows for precise target engagement, either into discreet or broad brain regions, unilaterally or bilaterally as needed. Third, the FUS mediated BBB opening is transient, with a therapeutic window of ~6 h, thus safely returning the BBB to its prior intact state. Fourth, FUS allows for administration of combinations of therapeutics. Several lines of evidence indicate that neurotrophic factors like GDNF can promote dopaminergic regeneration and may be required in addition to therapeutics that aid in targeting and clearing pathological asyn (270, 276). In support of this, FUS-mediated delivery of the neuroprotective agent D3 promoted neuronal survival and rescued memory deficits in a transgenic mouse model of Alzheimer's disease (AD) (277).

Nevertheless, some factors are still unknown and need to be addressed before FUS can be safely employed as a routine therapeutic strategy for brain targeting. For one, more data is required regarding the safety profile of BBB opening, and how it may be affected by age and disease onset. This is especially relevant for neurodegenerative diseases where the BBB integrity may be partially compromised (278). Secondly, more data on the long-term impact of chronic FUS treatment and associated neuroinflammation is warranted, although current evidence seems to suggest that FUS-mediated opening of BBB generates only a transient immune response and repeat administration does not affect BBB integrity or cause haemorrhagic complications (259). Additionally, data on how factors such as microbubble size, exposure parameters and acoustic energy can be used to fine-tune the size and duration of BBB opening may be important to determine ideal and personalized therapeutic approaches.

## Final Remarks

New tests to amplify and identify pathologically misfolded asyn in biopsy tissues are increasing in sensitivity (279–284) and may offer earlier detection of disease processes. In combination with prodromal clinical markers (285), these assays offer a critical opportunity to not only segregate synucleinopathy patients into distinct groups, but to also target asyn misfolding and accumulation using the precision tools discussed in this review. Initially, this will involve targeting the asyn assemblies with specific antibodies or by lowering asyn expression in specific brain regions to reduce the asyn substrate available for seeding. Ongoing improvements in viral vectors and gene delivery to the brain may offer less invasive and longer-term treatments to mitigate further pathological spread. Although much remains uncertain regarding age-related changes to other contributing factors such as proteostasis, inflammation, and oxidation, combinations of therapeutics targeting these ancillary pathways that regulate asyn processing could also be included as adjuncts to minimize neurodegeneration.

## Author Contributions

All authors listed have made a substantial, direct, and intellectual contribution to the work and approved it for publication.

## Funding

AT is supported by grants from the Canadian Institutes of Health Research (CIHR PJT180582) and the Weston Brain Institute (RR193223). SM received a postdoctoral fellowship from the Edmond J. Safra foundation. NPV is supported by the Rossy Foundation and Blidner Family Foundation. AH received a studentship from the Canadian Institutes of Health Research (CIHR-CGS-M).

## Conflict of Interest

SPS is a Sanofi employee and stockholder. The remaining authors declare that the research was conducted in the absence of any commercial or financial relationships that could be construed as a potential conflict of interest.

## Publisher's Note

All claims expressed in this article are solely those of the authors and do not necessarily represent those of their affiliated organizations, or those of the publisher, the editors and the reviewers. Any product that may be evaluated in this article, or claim that may be made by its manufacturer, is not guaranteed or endorsed by the publisher.
